# Strong Ferromagnetic Coupling between Co and Co^2+^ with Odd Electron (Anti)aromatic Radicals via Noncovalent Interaction

**DOI:** 10.1021/acs.jpca.5c01107

**Published:** 2025-05-09

**Authors:** Muskan, Debojit Bhattacharya, Suranjan Shil

**Affiliations:** † Manipal Centre for Natural Sciences, 76793 Manipal Academy of Higher Education, Manipal 576104, Karnataka, India; ‡ Kabi Sukanta High School (H.S.), Pati Colony, Siliguri, Darjeeling, West Bengal 734010, India

## Abstract

We have aimed to understand the interaction between odd electron aromatic or antiaromatic radicals with cobalt and their dipositive ion to understand the magnetic interaction between them. Density functional theory (DFT) along with the complete active space self consistent field (CASSCF) method has been used to calculate the magnetic exchange coupling constant (*J*) between the radical molecules and Co/Co^2+^. The DFT-calculated *J* ranging from 897 to 6060 cm^–1^ and 2534 to 18574 cm^–1^ for the CASSCF method signifies that the odd electron (anti)­aromatic-based magnetic molecules could be useful as strong low-dimensional magnetic materials. Frequency analysis reveals that some of the radicals behave as a transition-state structure in the absence of metal but become stabilized upon the addition of Co or Co^2+^. The noncovalent interaction (NCI) and electron localization function (ELF) analysis indicate that there is no covalent bonding between radicals and the metal. The absence of covalent bonding between the metal and radicals indicates direct ferromagnetic interaction between them. Aromaticity in the studied Co/Co^2+^–radical complexes has been evaluated using the nucleus independent chemical shift (NICS), harmonic oscillator model of aromaticity (HOMA), and gauge-including magnetically induced currents (GIMIC) analysis, revealing a complex, multidimensional nature of aromaticity. NICS(1) values indicated that the same ring exhibits both aromatic and antiaromatic behavior, depending on the spatial orientation of the metal center. The HOMA value shows a strong correlation with the magnetic exchange coupling constant (*J*), supporting a link between structural aromaticity and magnetic interaction. The aromaticity index GIMIC is not well correlated with other aromaticity indexes like NICS and HOMA. These observations highlight the need for multiple aromaticity descriptors to fully capture the complex aromatic character of these systems.

## Graphical abstract

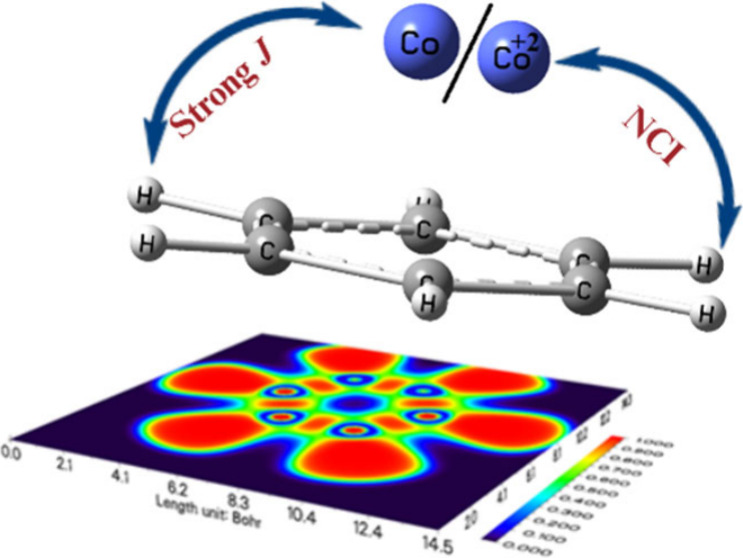

## Introduction

The age-old concept of aromaticity remains at the center as one of the most challenging and fascinating concepts in fundamental organic chemistry.^1–5^ The tuning of aromaticity brings intensive attention to experimentalists as well as theoreticians^6–11^ for its intriguing behavior. To study the constructive modification of aromaticity, some researchers have replaced carbon atoms of organic parent molecules with metal atoms to provide an avenue for the design of novel molecular structures.^7,8,12–21^ This kind of fragment replacement technique is very fruitful for designing antiaromatic skeleton organic species. As an example, one of the carbon atoms of four-membered antiaromatic cyclobutadiene is replaced by a silicon atom, resulting in the formation of a nonaromatic counterpart.^8^ On the contrary, replacement of the carbon atom at the bridgehead position in antiaromatic pentalene by a transition metal formed an aromatic species.^12,22^ It shows that the fragment replacement strategy in the antiaromatic skeleton brings about more aromatic character with extra stability. On the other hand, if a transition metal is used to replace aromatic fragments, then the formed organometallic compounds will try to keep its aromaticity.^23,24^ Replacement of the bridgehead carbon atom with an Os atom leads to an effective transformation from a planar aromatic condition to an antiaromatic one.^25^ Small-ring antiaromatic molecules are quite unstable and difficult to synthesize; however, some of them have been synthesized.^26^ Roy and co-workers^27^ have made a monoadduct and a diadduct of a four π-electron antiaromatic metal complex having Be as a metal ion.

Breslow and co-workers^28,29^ presented the concept of antiaromaticity during the mid-1960s, where the delocalization of electrons brings about destabilization. Over past few decades, researchers have used X-ray single-crystal diffraction as well as computational chemistry approaches to quantify aromaticity, among which the nucleus independent chemical shift (NICS) method^30,31^ is widely accepted. NICS is one of the best criteria of aromaticity and/or antiaromaticity, where absolute magnetic shielding is calculated at the center of the ring and here the NMR chemical shift conversion is reversed. The negative sign of the NICS value indicates aromaticity, and the positive sign indicates antiaromaticity. There are many theoretical treatments which have been applied to understand antiaromaticity, namely, spin-coupled theory,^32^ topological methods,^33,34^ etc.

Cyclopropenyl radical species have gained significant attention, particularly over the past decade.^35^ This radical is classified as aromatic;^35^ however, this classification remains subject to debate.^26^ The cyclobutenyl anionic radical is a 5π-electron radical system.^26^ As far as the cyclopentadienyl radical system is concerned, the complex molecular structure of this radical leads to extensive experimental and theoretical investigations. The Jahn–Teller distortion in the radical has been studied comprehensively.^36^ The cyclopentadienyl radical, characterized with a radical and two double bonds, can adopt numerous resonance structures that confirm the antiperiplanar hypothesis for electronic delocalization. The radical is equally distributed on both faces of the ring and across all five carbon atoms. According to the bent bond/antiperiplanar hypothesis (BBAH) model, this distribution indicates that the cyclopentadienyl radical is fully delocalized and can be classified as an aromatic radical.^37^ The cyclohexa 1,3-diene cationic radical is a 5π radical cation which shows antiaromatic character.^26^ The cycloheptatrienyl radical has been found to be stable despite having puckered geometry,^38^ but some reports show that its lower stabilization energy may be due to the antiaromaticity.^26^

In recent times, innovative magnetic materials have been designed for diverse practical applications such as spintronics,^39^ quantum electronics,^40^ and exhibiting photomagnetic behavior.^41,42^ We have recently worked on stable synthesized antiaromatic couplers in diradicals to understand their magnetic properties as well as other properties such as lowest unoccupied molecular orbital (LUMO) mediated magnetic exchange.^40^ Unlike the low magnetic exchange coupling constant (*J*) in aromatic coupler-based^43–45^ diradicals, we found very high *J* in the antiaromatic coupler-based diradicals.^13^ To get high *J* values in an organic diradical system, it is important to design and characterize prudently the magnetic molecular systems. Another popular way to increase *J* is to double the exchange coupling path^46^ when covalent bond interaction is concerned; however, when a noncovalent interaction has taken place, it is quite tricky to choose the parts of the total molecular entity. In this work, we have taken the (anti)­aromatic 3-carbon-containing cyclopropenyl radical to the 7-carbon-containing cycloheptatrienyl radical, which is a total of five cyclo­(anti)­aromatic radical structures (I to V) as the base (Figure 1) for our designed complexes. It should be mentioned here that one is an anionic (4-C containing) and another is a cationic radical organic moiety (6-C containing) and the remaining three have no additional charges (3-C-, 5-C-, and 7-C-containing moieties). The Co atom and Co^2+^ ion are placed above the plane of the basic organic (anti)­aromatic odd electron-containing fragments; therefore, a total of 10 complexes (5 structures with a Co atom and another 5 structures with a Co^2+^ ion) have been designed and investigated for characterization with possible applications considering their aromatic as well as magnetic characteristics.

**Figure 1:**
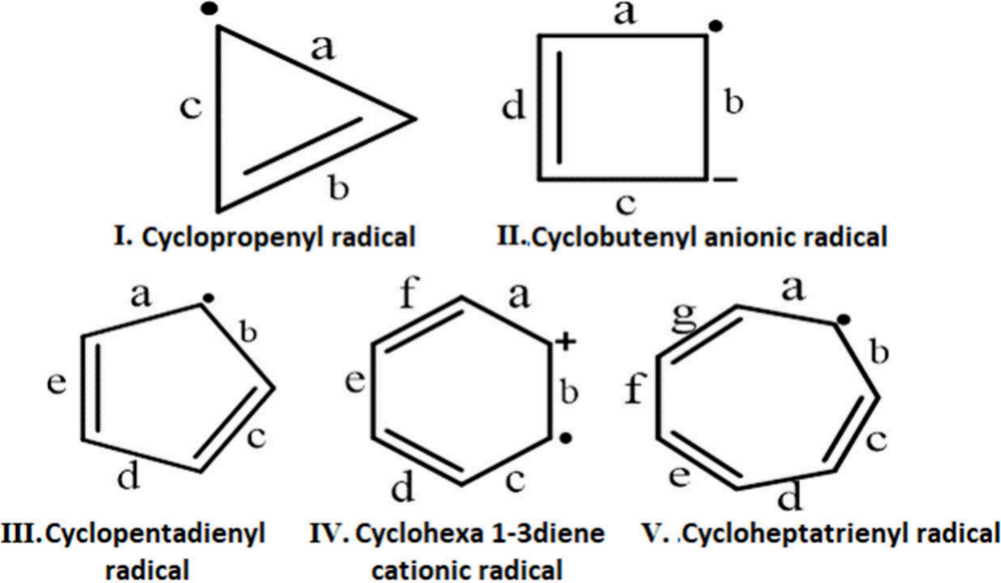
Structures of (anti)­aromatic radicals having a 3-carbon-containing cyclopropenyl radical to a 7-carbon-containing cycloheptatrienyl radical (I to V).^26^

## Theoretical and Computational Methods

The noncovalent interaction (NCI) analysis provides a tool for identifying noncovalent interactions using electron density and its derivatives. The NCI index is derived from a 2D plot of the reduced density gradient (*s*) and the electron density (ρ) where

1 $$s=\frac{\left|\nabla_{\rho }\right|}{2{\left(3\pi \right)}^{1/3}\rho^{4/3}}$$

in which the 4/3 term ensures that *s* is dimensionless.

Additionally, the NCI index classifies interactions as either attractive or repulsive based on the sign of the second-order density Hessian eigenvalue (λ2). To visualize this, a color code corresponding to the sign of (λ2)­ρ is often applied, linking 2D plots to 3D representations of the molecular system.^47^

To understand the noncovalent interaction in the complexes, we have calculated the NCI plot by using the NCIPLOT^48^ software. The visual look of the NCI plot is accomplished by using VMD software.^49^

The concept of Wiberg bond indices (WBI) was developed in the 1960s and further refined in the 1980s by Kenneth B. Wiberg.^50^ The WBI is derived from the density matrix of a molecule, specifically the off-diagonal elements of the atomic orbital density matrix, which indicates the extent of orbital overlap and electron delocalization between atoms. In our study, we performed natural bond orbital (NBO) analysis to calculate the WBI for each complex in both neutral and charged states. The WBI has been calculated using Gaussian 16 software.^51^

The magnetic criterion NICS^31^ has gained prominence as the predominant aromaticity probe due to its simplicity and effectiveness. The NICS has been calculated using the gauge independent atomic orbital (GIAO)^52^ methodology with the B3LYP functional and EPR-II basis set employing the Gaussian 16 software.^51^ The NICS value reflects the absolute shielding, expressed as a negative quantity, at a specific point within a molecular system. A more negative NICS value signifies greater aromaticity, corresponding to an intensified ring current induced by π electrons in aromatic compounds. In this study, NICS values were computed for all bare radicals, with neutral and charged metal centers. To minimize the influence of σ aromaticity and emphasize the contribution of the π-electron ring current, NICS values were determined with the probe placed at the center of the ring (NICS (0)), 1 Å above the ring center toward the metal denoted as NICS (+1) and 1 Å below the ring center (opposite to the metal atom or its ion) denoted as NICS (−1).

The Heisenberg–Dirac–van Vleck spin Hamiltonian is commonly used to express the magnetic exchange interaction between magnetic sites 1 and 2, as shown below:

2 $$Ĥ=-2J{Ŝ}_{1}{Ŝ}_{2}$$

Here, *J* denotes the magnetic exchange coupling constant, while *Ŝ*_1_ and *Ŝ*_2_ represent the spin angular momentum operators for these centers. A positive *J* corresponds to ferromagnetic coupling, indicating a preference for parallel spins. Conversely, a negative *J* signifies antiferromagnetic interaction, favoring antiparallel spins. For a diradical with one unpaired electron at each radical site, *J* can be defined as

3 $$E_{S=1}-E_{S=0}=-2J$$

The broken symmetry (BS) approach introduced by Noodleman^53^ in the DFT framework offers a more computationally feasible route to evaluate *J*. Various formalisms^54–59^ have been developed to evaluate *J* using unrestricted spin-polarized broken-symmetry (BS) solutions, tailored to the degree of magnetic interaction between the two magnetic sites. The method by Ginsberg,^54^ Noodleman,^55^ and Davidson^56^ is applicable when interactions are weak, whereas the formalisms from Bencini^57^ and Ruiz^58^ are better for the strong overlap between orbitals (eq 4).

4 $$J_{B}=\frac{\left(E_{BS}-E_{T}\right)}{S_{\max}\left(S_{\max}+1\right)}$$

Yamaguchi’s^59^ equation is good for both weak and strong interactions (eq 5).

5 $$J_{Y}=\frac{\left(E_{BS}-E_{T}\right)}{{\left\langle S^{2}\right\rangle }_{T}-{\left\langle S^{2}\right\rangle }_{BS}}$$

Here, *E*_BS_ and *E*_T_ represent the total energies of the broken-symmetry (BS) singlet and triplet states, respectively, while ⟨*S* ^2^⟩_T_ and ⟨*S* ^2^⟩_BS_ represent average spin-square values for triplet and BS states, respectively.

To verify the consistency of DFT results, we have used the single-point complete active space self-consistent field (CASSCF) (number of electrons = 14 and number of orbitals = 10) calculation on the density functional theory (DFT) optimized geometry.

Ziółkowska and Witwicki^60^ studied the magnetic properties of Cu complexes by DFT and CASSCF methods, and they found that the DFT and CASSCF methods are not well correlated in terms of the values of the magnetic exchange coupling constant compared to the experimental values. It has been shown that the CASSCF calculations with minimal and extended active spaces are insufficient to quantitatively reproduce the magnitude of the exchange coupling observed experimentally. The small active space is inadequate to capture the physics of the spin coupling, and the method is unable to recover dynamic electron correlation. Therefore, we choose 14 electrons and 10 orbitals to consider all of the 3d and 4s electrons of Co and the unpaired electron of the radical.

For a system to exhibit single-molecule magnet (SMM) behavior, a significantly negative zero-field splitting (ZFS) parameter (*D*) value is both expected and necessary. Computationally, ZFS parameters can be derived using perturbative approaches in DFT.^61^ The spin–orbit coupling contribution to ZFS is determined through a perturbative theoretical approach within the framework of unrestricted Kohn–Sham theory.^62,63^ A simplified equation can be derived to relate the axial ZFS parameter (*D*), the rhombic ZFS parameter (*E*), and the anisotropy tensor components (*Y*_*xx*_, *Y*_*yy*_, *Y*_*zz*_) as follows

6 $$H_{ZFS}=D\left[{S_{Z}}^{2}-\frac{1}{3}S\left(S+1\right)\right]+E\left[{S_{x}}^{2}-{S_{y}}^{2}\right]$$

where *S* represents the total spin quantum number and *S*_*x*,*y*,*z*_ represents the spin matrices. To evaluate the magnetic properties of a system, it is crucial to determine both the magnitude and the sign of *D*. A positive *D* value indicates easy-plane anisotropy which is not useful as an SMM. Conversely, a negative *D* value is essential for a system to exhibit SMM behavior.^64^

All of the radicals (without metal, with neutral metal, and with charged metal) were first optimized with the B3LYP^65,66^ hybrid density functional theory in combination with the def2-TZVP basis set^67^ and D3BJ^68,69^ correction. These calculations were performed using ORCA 5.0.1 software.^70^ Although the B3LYP functional failed for metal systems,^71^ in our recent study^72^ we have shown that B3LYP works well for the magnetic exchange coupling constant of metal complexes, which correlates well with the other previous studies.^58,73^ It has been shown that for DFT the def2-TZVP basis set would reach the complete basis set limit with reasonable computational cost.^67,74^ We have computed the harmonic oscillator model of aromaticity (HOMA) index and electron localized function (ELF) using multiwfn^75^ software.

The gauge-including magnetically induced current (GIMIC)^76,77^ analysis has been carried out using GIMIC code from the formatted check point file of the Gaussian program. The GIMIC plots were made with the ParaView program.^78^

## Results and Discussion

There are studies^79^ in which it has been found that the presence of 6 π-electrons (see Clar’s theory of the aromatic sextet^80^ and extended Clar theory for polyacenes and beyond,^81^) makes a ring stable. In the upcoming discussions, panels A and B contain the complexes of Co and Co^2+^, respectively. Five-membered (radical III) and seven-membered (radical V) ring radicals shows negative frequency after optimization, which indicates their instability in the radical form. However, we obtained a negative frequency for the complexes of the cyclopropenyl radical (I) with the Co metal and the cyclopentadienyl radical (III) with Co^2+^. Although the Co doublet and quartet states are very close in energy, in this work we consider the doublet state of cobalt in the complexes, which gives the lowest-energy ground state of all of the complexes except the complex of Co^2+^ and the cyclobutenyl anionic radical (energy values are given in the Supporting Information, Table S23). The other first-row 3d transition metals such as Ni and Fe produce a low-spin ground state with the radicals considered in this work (Tables S24 and S25). This behavior can be explained by the interaction between the metal and radical orbitals. In the high-spin configuration, the electrons occupy the higher-energy antibonding orbitals, particularly *d*_*z* ^2^_ and *d*_*x* ^2^ *–y* ^2^_. Occupation of these orbitals leads to elongation of the metal–ligand bond distances, resulting in a destabilized geometry and overall unfavorable metal–radical interactions. In contrast, the low-spin configuration avoids occupying these antibonding orbitals, thereby maintaining shorter metal–ligand bonds and stronger orbital overlap with the radical. As a result, low-spin states are energetically preferred for Ni and Fe in these complexes and the high-spin solutions often do not converge.

### Bonding Analysis

#### Noncovalent Interactions (NCI)

For all of the Co complexes (Figure 2, panel A), the plots demonstrate regions with negative λ2 values, represented in blue, indicating the presence of strong attractive interactions and positive λ2 values, represented in red, indicating the presence of repulsive interactions. The strong attractive interactions are typically associated with hydrogen bonding, π–π stacking, etc., suggesting that these forces play a significant role in the stabilization of the neutral complexes and that the repulsive interactions are likely due to the steric interaction or Pauli repulsion, which may balance the attractive forces to some extent. Importantly, no sharp peaks corresponding to strong attractive interactions (typically observed at low *s* values) were observed in any of the neutral complexes. Instead, the NCI plots showed broad regions of positive λ2, suggesting a more distributed presence of repulsive interactions. In particular, complex II displayed regions of both positive and near-zero λ2 values, and these values are shown in green, indicating weak van der Waals-type interactions where there is neither significant attraction nor repulsion. This complex featured weak interactions on both sides (positive and negative λ2) of the molecule, unlike complex V, which shows weak interactions on only one side (positive λ2).

**Figure 2:**
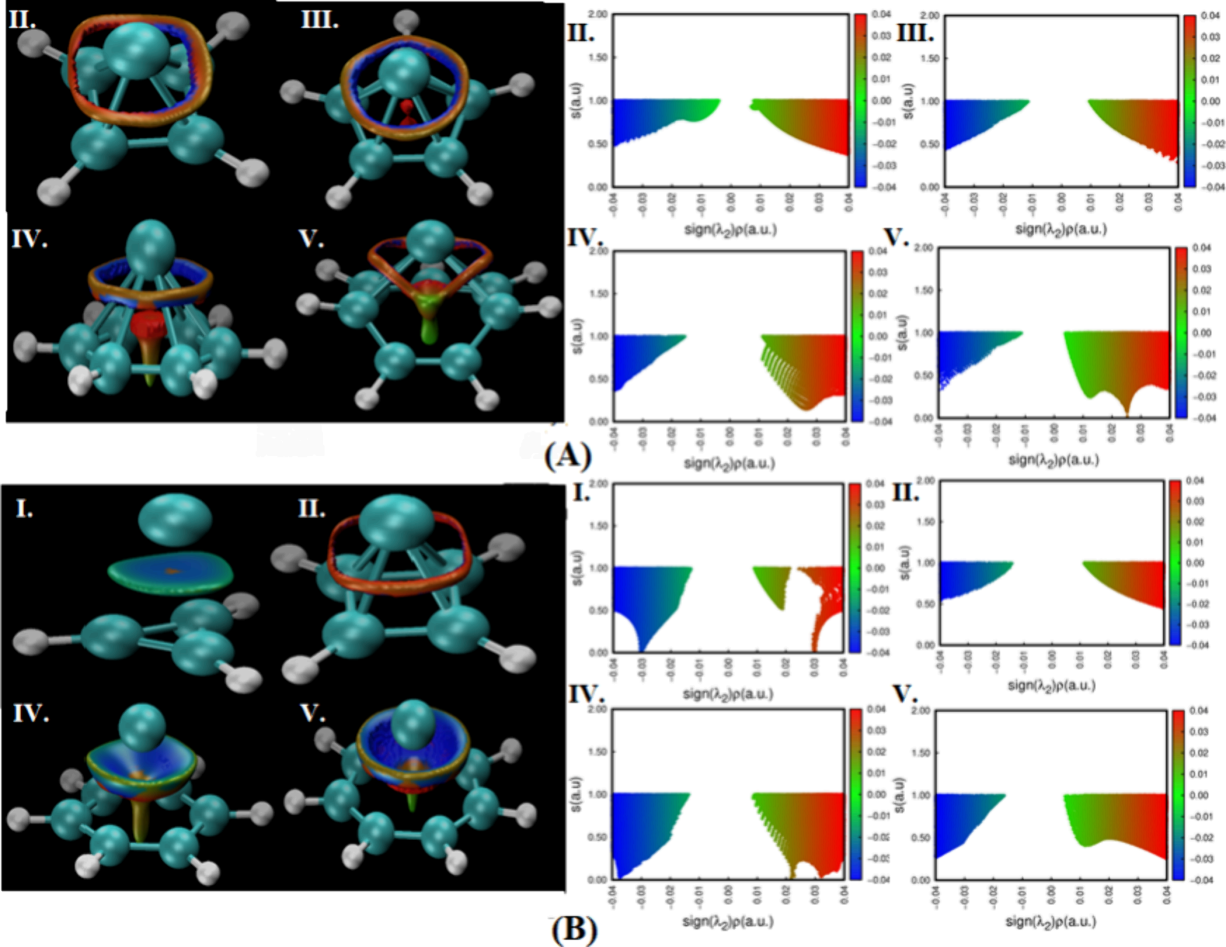
NCI plot of all of the complexes. (Panel A corresponds to neutral complexes, and panel B corresponds to the charged complexes.)

The Co^2+^ complexes (Figure 2, panel B) show a pattern similar to that of Co complexes, in which all complexes showing blue regions correspond to negative λ2 values, with red regions related to positive λ2 values. Complex V exhibits green regions on one side (positive λ2) of the molecule, corresponding to near-zero λ2 values, indicating weak van der Waals interactions. In contrast, other complexes displayed green regions as well but not near-zero values, signifying weaker interactions compared to those of complex V. Note that complex I and complex IV each exhibited one sharp peak at low *s* values corresponding to negative λ2 values. These peaks suggest the presence of strong localized attractive interactions, which are absent in the other charged complexes. Broad regions of positive λ2 values were observed in all Co^2+^ complexes, indicating areas of repulsive interactions.

Overall, NCI analysis reveals that both neutral and charged cobalt complexes are characterized by a balance of attractive and repulsive interactions. There is no evidence of covalent bonding between the metal and the radical rings.

#### Wiberg Bond Index (WBI)

Our findings reveal that for complexes IV and V, the WBI for Co decreases when moving from the neutral to the charged state, suggesting a reduction in covalency in the charged state. In contrast, for complex II, the WBI for Co increases upon charging, indicating stronger bonding or interaction in the charged state, as illustrated in Figure 3.

**Figure 3:**
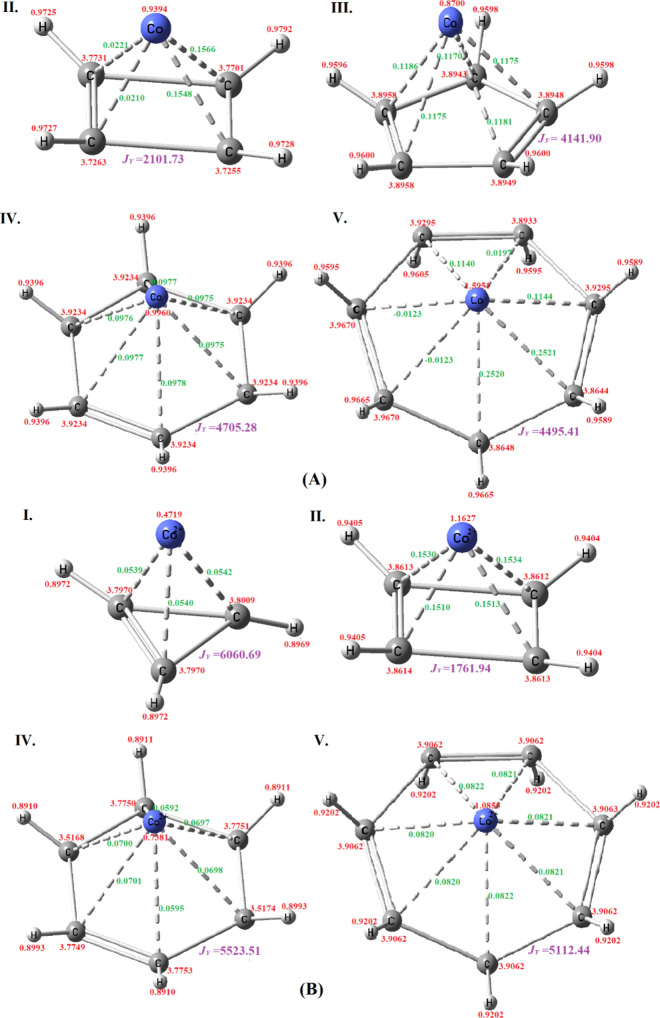
WBI and atom–atom overlap weighted bond order are indicated by red and green digits, respectively. The purple color value indicates the value of magnetic exchange coupling constant *J*_*Y*_ in cm^–1^. (Panel A corresponds to neutral metal complexes, and panel B corresponds to the charged metal complexes.)

If we compare the WBI of Co between the neutral and charged states of all of the complexes, we can see that where the WBI of Co is higher, the *J* value is lower for that complex as shown in Figure 3. This relationship suggests that a higher WBI, indicative of stronger covalent interactions, corresponds to lower *J* values. This is consistent with the understanding that the exchange coupling constant (*J*) typically requires unpaired electrons or radical centers; thus, increased covalent interactions imply electron pairing, which reduces the magnitude of *J*.

The atom–atom overlap weighted bond order between Co and C of all of the neutral complexes is unequal except for complex IV where the bond orders are equal. However, in charged complexes, this trend is opposite; all of the complexes have equal atom–atom weighted bond orders except for complex IV where the bond orders are different. This suggests that the complex with six-membered rings has different behavior compared to that of the other radicals studied here. When the Co and six-membered ring cation come together, the radical accepts one electron from the Co and tries to become aromatized, which can be seen in the C–C bond lengths where all of the lengths are equal in the neutral complex (Table S2). Hence, the atom–atom weighted bond orders are equal.

#### Electron Localization Function (ELF)

The ELFs^82^ of all of the complexes are plotted in Figure 4. The high value of electron localization is denoted by red, and the chemical bonds are described by irregular localization denoted by orange. The localized electrons are represented in blue.^83^ The high values of ELF around the Co atoms show highly localized bonding and nonbonding electrons. The blue region between the Co and C atoms of the rings indicates the localized electrons and confirms the considerable electrostatic interaction between the Co atom and the C ring. The ELF plots indicate that Co/Co^2+^ have strong electrostatic interactions with the carbon rings, which form the nonbonded stable complexes.

**Figure 4:**
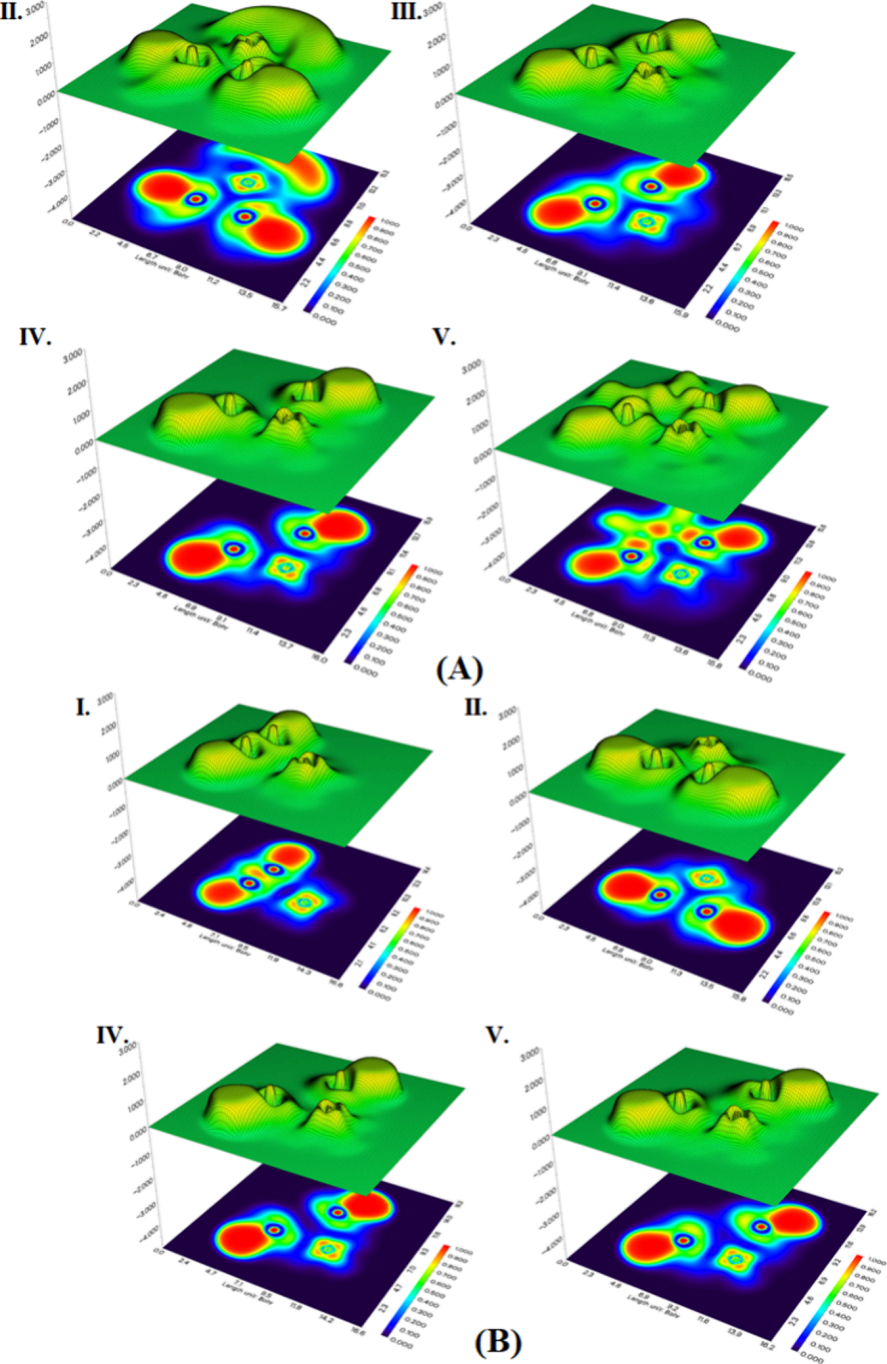
2D map of η­(r) function for the molecular plane defined by the C–Co–C atoms. The ELF has been calculated using the B3LYP/def2-TZVP level of theory. (Panel A corresponds to neutral metal complexes, and panel B corresponds to the charged metal complexes.)

#### Aromaticity Indexes NICS and HOMA

Aromaticity is a multidimensional property^84^ with no direct linear correlation between different aromaticity indices, as supported by our study (Table 1). Radical I, in the absence of a metal center, exhibits negative NICS values, indicating aromaticity, while its negative HOMA value suggests antiaromaticity. However, after complexation with Co^2+^, the radical maintains aromaticity with more consistent results, as evidenced by negative NICS values and a shift in HOMA to a positive value. Radical II shows antiaromaticity without a metal, as indicated by both the NICS and HOMA values. After complex formation with a metal atom or ion, it displays mixed aromatic and antiaromatic behavior due to the heterogeneous electron distribution, with NICS values, while HOMA continues to suggest antiaromaticity. For radical III, only one stable complex with Co was obtained, which exhibits antiaromaticity according to HOMA, while NICS values show mixed behavior, attributed to different electron densities on both sides of the ring. Radical IV shows antiaromaticity according to NICS and aromaticity according to HOMA in its free state. In the Co complex, the radical exhibits a negative NICS (1) value, strongly confirming its aromatic nature.^85^ The bond indices for all ring carbon and hydrogen atoms are perfectly equal, indicating a uniform electron distribution (Figure 3) in the Co complex. In the Co^2+^ complex, this uniformity is disrupted, resulting in mixed aromatic and antiaromatic behavior, as indicated by the NICS values. However, the HOMA values of the charged and neutral complexes are shown as aromatic. Radical V is unstable without a metal center. The neutral complex is shown as aromatic, as NICS values suggested. The charged complex exhibits contradictory aromaticity predictions, with NICS values indicating mixed aromatic and antiaromatic behavior. The HOMA values predicted antiaromaticity for both complexes. This inconsistency is likely due to different electron distributions on both sides of the radical ring of the complexes.

**Table 1:** NICS Values of the Complexes at B3LYP/def2-TZVP with GIAO Methodology and HOMA Values from the Optimized Structures at the B3LYP/def2-TZVP Level of Theory^a^

		Radical			Radical-Co complex				Radical-Co^2+^ complex			
Sl. No.	Complex	NICS (0)	NICS (1)	HOMA	NICS (0)	NICS (+1)	NICS (−1)	HOMA	NICS (0)	NICS(+1)	NICS (−1)	HOMA
I	Cyclopropenyl radical	–19.47 (−6.68)	–1.93 (−3.56)	–0.336					–46.08 (−51.76)	–75.56 (−65.43)	–63.95 (−35.85)	0.980
II	Cyclobutenyl anionic radical	14.87 (−22.71)	15.34 (7.35)	–1.083	–32.16 (−78.88)	19.67 (4.54)	–28.25 (8.88)	–0.644	–558.30 (−1585.15)	–275.20 (−835.28)	93.81 (309.17)	–2.180
III	Cyclopentadienyl radical				49.10 (66.92)	44.08 (26.35)	–214.26 (59.90)	–130.041				
IV	Cyclohexa1-3diene cationic radical	26.02 (83.81)	17.31 (55.12)	0.713	49.97 (173.13)	–46.89 (−236.28)	–190.39 (−437.50)	0.866	–10.58 (−22.03)	5.94 (−77.84)	–25.55 (−45.85)	0.250
V	Cycloheptatrienyl radical				–34.21 (−13.97)	–22.47 (−52.10)	–14.98 (−31.41)	–332.877	16.56 (24.67)	18.02 (−33.1642)	–18.15 (−6.26)	–399.197

a The values in the parentheses are the values of NICS_*zz*_.

In many of our previous studies,^43,86^ we observed that when aromatic couplers were used, NICS and HOMA values followed the same trend. However, when antiaromatic couplers were employed, we found that NICS and HOMA exhibited opposite trends.^40^ In this work, we observe a mixed trend between NICS and HOMA in predicting the aromaticity of our designed complexes (Table 1). For example, when calculating NICS(−1) for the same species, such as Co^2+^ complexes II and V, we notice that NICS(0) and NICS(1) values show opposing trends. In a few cases, such as Co complexes III and IV, the NICS(0) and NICS(−1) values exhibit opposite trends. Furthermore, we found that NICS(1) and NICS(1)­zz values also show opposing trends in certain instances, such as for radicals II and III with a Co atom in NICS(−1) and radical IV with Co^2+^ in NICS­(+1). This suggests that the presence of a metal and its ion disturbs the electron distribution around the ring, causing the NICS values to appear inconsistent, depending on the position of the dummy atoms used for the calculations.

Among the Co^2+^ complexes, complex I exhibited the highest positive HOMA value, correlating with the enhanced aromaticity. The HOMA value of radical IV and its Co^2+^ complex strongly supports their aromatic nature, with equalized C–C bond lengths providing further confirmation (Supporting Information Table S2). These observations collectively demonstrate that metal centers and their ions induce significant changes in the aromaticity of the studied radicals, often resulting in mixed trends for aromaticity indices, such as NICS and HOMA.

#### Gauge-Including Magnetically Induced Currents (GIMIC)

The GIMIC plots of all of the complexes are given in Figure 5. Neutral complexes II and V have paratropic ring currents whereas complexes III and IV have diatropic ring currents. However, charged complexes I and V have diatropic ring currents and complex II has a paratropic ring current. It has been observed that a transition from diatropic (aromatic) to paratropic (antiaromatic) ring current upon one-electron oxidation or reduction in benzene radical ions maintains energetic stability in both states.^87^ Interestingly, charged complex IV has a ring current perpendicular to the ring, and it has two components, paratropic and diatropic ring currents. Foroutan-Nejad^87^ investigated the validity of the paratropic ring current as an aromaticity index in monocyclic hydrocarbon, and he showed that the aromaticity of a compound depends on the chosen computational methodology and descriptor. We have analyzed the aromaticity indexes NICS, HOMA, and GIMIC, and none of these parameters correlate well with each other. For some complexes, they are consistent. For example, charged complex I has positive HOMA, negative NICS, and a diatropic ring current, suggesting that this is an aromatic complex. However, in charged complex V the HOMA suggests it is an antiaromatic, but GIMIC suggests that it is an aromatic. These properties contradict each other. This analysis proves that the aromaticity cannot be quantified with a single descriptor and is multidimensional in nature.

**Figure 5:**
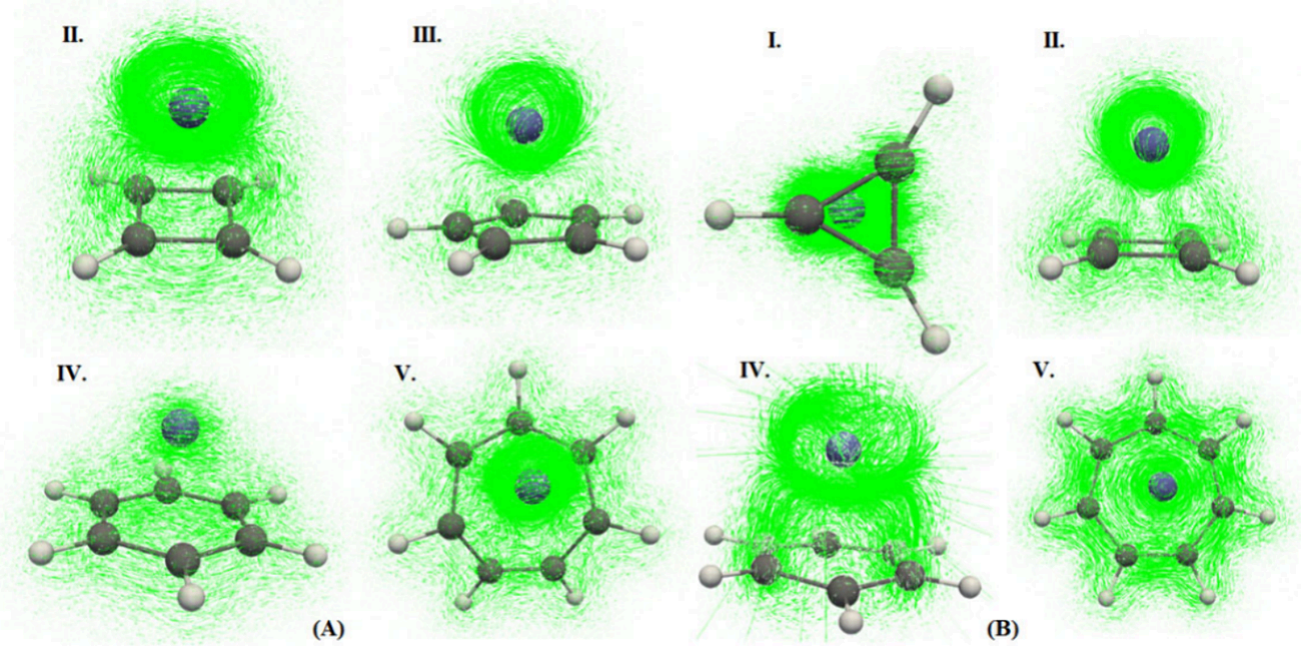
GIMIC plot of all of the complexes at the B3LYP/def2-TZVP level of theory. (Panel A corresponds to neutral metal complexes, and panel B corresponds to the charged metal complexes.)

### Magnetic Exchange Coupling Constant

The *J* values of all of the complexes are represented in Table 2 at different levels of theory. In all cases, the value of *J* is positive; hence, in each case, the triplet is the ground state. In DFT methodologies, we have used two methods to calculate the value of *J* as already stated in the theoretical and computational details. For the *J*_Y_ and *J*_B_ values down the series in those complexes with a Co atom, we have found that from complex II to III the *J* value increased by more than two times. When moving from complex III to IV, we got an increment of *J* values by almost 15%. Whereas in the case of complex V, the *J* values decreased by 1–5%. We observe an increment in *J* for neutral Co complex II to III to IV and a decrement from complex IV to V because of the stability of the rings. The value of *J*_Y_ is always higher than the value of *J*_B_ down the series as determined by DFT methods for both (anti)­aromatic radical complexes with the Co atom and with its dipositive ion, whereas in the CASSCF method *J* values are always found to be higher down the series in the corresponding cases. Nevertheless, when we are dealing with the same with Co^2+^ we observed that the *J* values increased from complexes II to IV and decreased from complexes IV to V as determined by DFT methods. In the CASSCF method, an increment of *J* is observed in the case of complex V. Another point to be noted here is that *J* values for complex I are highest among all the cases using the DFT method with higher HOMA values (Table 1). If we compare HOMA with the *J* values of DFT methods, we find that a high *J* value is correlated with a high value of HOMA. This correlation suggests that there is a relationship between *J* values and the structural aromaticity index. The magnetic exchange coupling constant values of all of the complexes suggest that these complexes can be used as very strong low-dimensional ferromagnetic materials.

**Table 2:** Magnetic Exchange Coupling Constant (*J*, cm^–1^) of the Complexes at the B3LYP/def2-TZVP Level of Theory

		Radical-Co complex			Radical-Co^2+^ complex		
	Complex	*J*_Yamaguchi_	*J*_Bencini_	*J*(CASSCF)	*J*_Yamaguchi_	*J*_Bencini_	*J*(CASSCF)
I	Cyclopropenyl radical complex	-	-	-	6060.69	3069.22	15077.91
II	Cyclobutenyl anionic radical complex	2101.73	897.14	2598.58	1761.94	1204.37	2534.93
III	Cyclopentadienyl radical complex	4141.90	2065.58	14737.72	-	-	-
IV	Cyclohexa1–3diene cationic radical complex	4705.28	2401.23	15020.84	5523.51	2976.95	13229.93
V	Cycloheptatrienyl radical complex	4495.41	2377.19	9828.07	5112.44	2691.13	18574.14

### Molecular Orbitals

The energy levels and shapes of the singly occupied molecular orbitals (SOMOs) and the lowest unoccupied molecular orbital (LUMO) play a key role in shaping the molecule’s magnetic behavior and transport properties. According to Hund’s rule, when the energy gap between two SOMOs is relatively small, a triplet ground state is typically favored. Again, Hoffmann et al.^88^ in 1968 using extended Hückel calculations provided an empirical rule stating that if Δ*E*_SOMO–SOMO_ is less than 1.5 eV then the two electrons in the nonbonding SOMOs will try to favor parallel spin configuration, which results in a triplet ground state.^88,89^ On the other hand, Constantinides et al.^90^ have found that if Δ*E*_SOMO–SOMO_ is greater than 1.3 eV then the electrons will occupy bonding conditions in two nondegenerate SOMOs in the case of heteropolyacenes. The energy gap between the orbitals is presented in Figure 6. We have found that in our complexes Δ*E*_SOMO–SOMO_ values do not exceed 1.5 eV in 5 out of 8 cases. Nonetheless, in the remaining 3 cases we have also found that Δ*E*_SOMO–SOMO_ is more than 1.5 eV (up to 2.6 eV), although they are showing a triplet ground state. We can see that in Co complex II the SOMO–SOMO gap is 0.34 eV with a *J* value of 2101.73 cm^–1^ whereas in the Co^2+^ complex the gap increases to 2.65 eV with lower *J* (1761.94 cm^–1^). This reason is also applied to complex V. However, complex IV gives opposite trends where the Co complex gives a lower *J* value (*J* = 4705.28) with a zero SOMO–SOMO gap. On the other hand, the Co^2+^ complex gives a higher *J* value (*J* = 5523.51) with a SOMO–SOMO gap of 1.56 eV. This could be explained with the help of the HOMO–LUMO gap and the coefficient of SOMOs and LUMO. All of the SOMOs have significant contributions from metal except for charged complex IV. In complex IV, if we look at the HOMO–LUMO gap the Co complex has a HOMO–LUMO gap of 4.93 eV, but in the Co^2+^ complex, the gap is 2.06, which is a significant reduction from the neutral complex. Moreover, in the charged state, the SOMOs are in the radical ring with a very negligible contribution from the metal. This causes the complex to resemble organic radicals, where the HOMO–LUMO gap is one of the determining factors in the strength of magnetic coupling constant *J*.^91^

**Figure 6:**
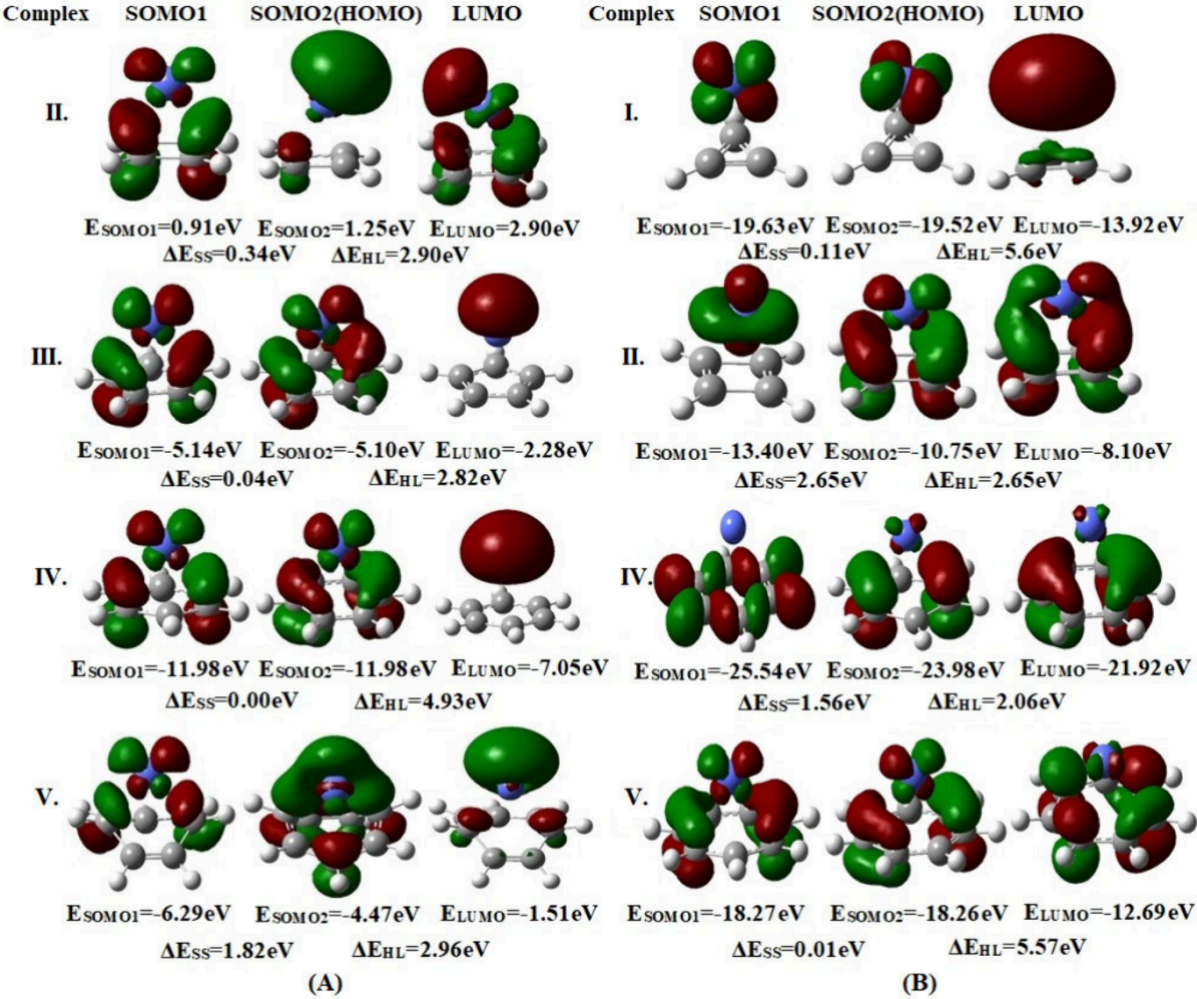
Molecular orbitals (SOMO-1, SOMO-2, and α-LUMO) and energies with SOMO-2–SOMO-1 gaps (Δ*E*_SS_) and HOMO–LUMO gaps (Δ*E*_HL_) for radical complexes at the B3LYP/def2-TZVP level of theory in the high-spin (triplet) state. The isovalue for the MO plots is set at 0.05. Blue, gray, and white atoms represent the cobalt, carbon, and hydrogen atoms, respectively. (Panel A corresponds to neutral complexes, and panel B corresponds to the charged complexes.)

If we look at the symmetries of the SOMOs of all of the complexes in Figure 6, we can observe that there are low-energy gaps between the symmetric SOMOs. For example, Co complex IV has zero SOMO–SOMO energy gap, whereas the nonsymmetric SOMOs have a higher energy gap such as that for Co^2+^ complex II (SOMO–SOMO energy gap of 2.65 eV). If we consider the HOMO and LUMO of neutral and charged complex II, we can see that the HOMO and LUMO have most of the contribution from the Co atom in the neutral complex; however, for the charged complex the symmetry is broken. This explains the high *J* value in the neutral complex. In the case of complexes IV and V, most of the contributions for HOMO and LUMO are from the radicals in the charged state and have higher *J* values compared to those of their neutral counterpart.

### Zero-Field Splitting

The ZFS parameter in high-spin molecular systems provides insight into their potential as single-molecule magnets. A significant negative value of ZFS parameter D suggests that the system could function effectively as a single-molecule magnet. However, most molecular systems, despite having a high-spin ground state, exhibit either positive or only slightly negative D values. As a result, identifying a molecular system with both a high-spin ground state and a significantly negative D remains a challenge. In this study, we calculated the zero-field splitting values for all of the radical complexes (Table 3).

**Table 3:** Zero-Field Splitting Parameter of the Complexes at the B3LYP/def2-TZVP Level of Theory

		Radical-Co complex		Radical-Co^2+^ complex	
Sl. No.	Complex	*D* (cm^–1^)	*E*/*D*	*D* (cm^–1^)	*E*/*D*
I	Cyclopropenyl radical complex	-	-	–4.02	0.01
II	Cyclobutenyl anionic radical complex	–16.82	0.08	1.88	0.07
III	Cyclopentadienyl radical complex	11.70	0.00	-	-
IV	Cyclohexa1–3diene cationic radical complex	9.01	0.00	10.51	0.13
V	Cycloheptatrienyl radical complex	8.68	0.02	9.01	0.00

Co^2+^ complex I has a negative D (−4.02 cm^–1^) value and a small E/D value, and Co complex II has a large negative D (−16.82 cm^–1^) value. This makes these complexes strong candidates for application in single-molecule magnets. Notably, Co^2+^ complex I also has the highest *J* value per the DFT calculation and the highest HOMA value. This indicates that this complex is an ideal candidate for a low-dimensional aromatic ferromagnet.

### Spin Density Analysis

The dominance of the triplet state is further indicated by spin density plots as already found in many cases.^43–45^ From Figure 7, it is seen that a large spin density contribution is shown by Co and Co^2+^ as compared with the (anti)­aromatic organic entity in each complex. An increase in *J* values from one complex to another happens when spin density is increased in the Co atom of the complexes; however, for the Co^2+^ complexes the reverse trend is observed due to charge transfer from the (anti)­aromatic radical to the charge-deficient Co^2+^ ions.

**Figure 7:**
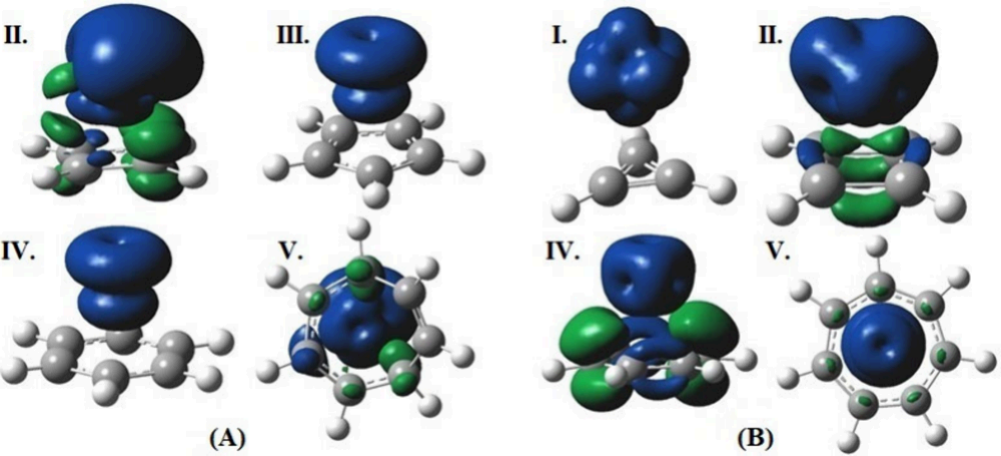
Spin density plot of all of the complexes in their triplet state at the UB3LYP/def2-TZVP level of theory. (Panel A corresponds to neutral complexes, and panel B corresponds to the charged complexes.)

## Conclusions

In this study, we have investigated the aromaticity and magnetic properties of five (anti)­aromatic radicals when complexed with a Co atom and a Co^2+^ ion. We have observed that the interaction of antiaromatic radicals with a Co atom or a Co^2+^ ion leads to strong ferromagnetic complexes. Our findings reveal that metal atoms transform antiaromatic molecules into aromatic ones or enhance their stability, depending on the metal’s charge state and the nature of the radical. The magnetic exchange coupling constants are well correlated with the structural aromaticity index HOMA. NCI analysis highlights the coexistence of strong attractive and repulsive noncovalent interactions, emphasizing the delicate balance of forces that regulate these systems. Furthermore, ELF plots confirm strong electrostatic interactions between the Co and the radical, supporting the noncovalent yet stable nature of these complexes. The GIMIC analysis provides additional insights into the aromatic character by revealing both diatropic and paratropic ring currents, depending on the complex and charge state of the complexes. Overall, our work highlights the potential of metal–(anti)­aromatic organic radical complexes for designing novel magnetic materials with tailored aromatic and electronic properties, providing insights into the development of advanced molecular magnets and quantum materials.

## Supplementary Material



## Notes

Optimized coordinates of all of the complexes; table of bond lengths; Wiberg bond index; atom–atom overlap index; distance between cobalt and the rings; ELF plot of the complexes; plot of *J* vs NICS and HOMA; and energy values of Fe, Ni, and Co complexes (PDF)
