# Supplementary material for: Strong Ferromagnetic Coupling between Co and Co2+ with Odd Electron (Anti)aromatic Radicals via Noncovalent Interaction
Source: J Phys Chem A. 2025 May 9;129(20):4426–37. doi: 10.1021/acs.jpca.5c01107 (PMC12105019; doi:10.1021/acs.jpca.5c01107)
Supplement: Supplementary file 1 [file jp5c01107_si_001.pdf]

Strong Ferromagnetic Coupling between Co and Co^{2+} with Odd Electron (Anti)aromatic Radicals via Noncovalent Interaction

Muskan[†], Debojit Bhattacharya[‡], Suranjan Shil[†*]

[†]Manipal Centre for Natural Sciences, Manipal Academy of Higher Education, Manipal 576104, Karnataka, India, [*]Email: suranjan.shil@manipal.edu, Phone: **+91-820-2923580**

[‡]Kabi Sukanta High School (H.S.), Pati Colony, Siliguri, Darjeeling, West Bengal, 734010, India

Supporting Information

Structural information

Total Covalent Radius of Co and C= 1.99 Å

Total Vander Waals Radius of Co and C= 4.17 Å

Table S1. The distance between Co and C

Complexes						
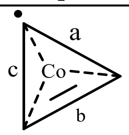	2.02389	1.99922	2.00677	-	-	-
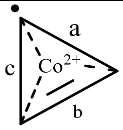	2.54596	2.54635	2.53882	-	-	-
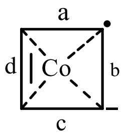	2.06767	2.18280	2.17978	2.06524	-	-
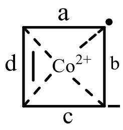	2.10568	2.10672	2.10988	2.10887	-	-
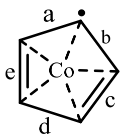	2.14782	2.15018	2.15142	2.15024	2.15303	-
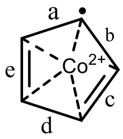	2.26566	2.28057	2.25878	2.21272	2.21668	-
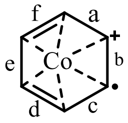	2.20109	2.20004	2.20029	2.20050	2.20030	2.20106

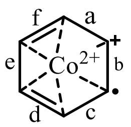	2.50248	2.56244	2.50077	2.50149	2.56422	2.50310
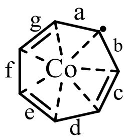	2.10652	2.11520	2.83078	2.83098	2.11482	2.10515
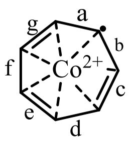	2.30231	2.30183	2.30197	2.30217	2.30188	2.30208

Table S2. C-C Bond lengths of molecules without metal, with metal (Neutral and Charge)

Molecules	a	b	c	d	e	f	g
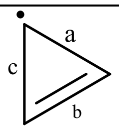	1.45528	1.30801	1.45600	-	-	-	-
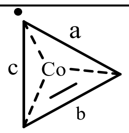	1.41492	1.41958	1.41568	-	-	-	-
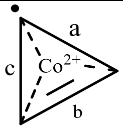	1.37920	1.37925	1.37884	-	-	-	-
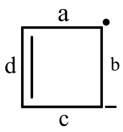	1.51489	1.39457	1.51509	1.39464	-	-	-
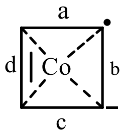	1.42925	1.51393	1.42996	1.46672	-	-	-
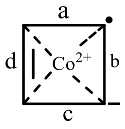	1.37983	1.54502	1.38041	1.54476	-	-	-
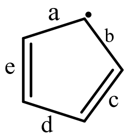	1.36608	1.43337	1.43310	1.36624	1.47899	-	-
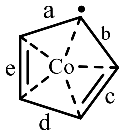	1.42410	1.42796	1.42773	1.42403	1.43043	-	-

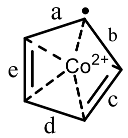	1.39562	1.49428	1.39563	1.45039	1.45110	-	-
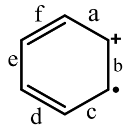	1.42537	1.36483	1.42552	1.42537	1.36483	1.42552	-
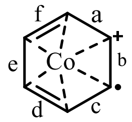	1.41076	1.41083	1.41039	1.41079	1.41082	1.41037	-
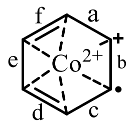	1.45381	1.39741	1.45376	1.45370	1.39739	1.45369	-
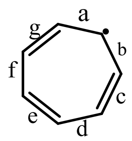	1.35779	1.43112	1.38662	1.39131	1.42707	1.35977	1.44802
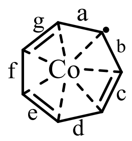	1.42236	1.41845	1.41866	1.42181	1.48136	1.33375	1.48153
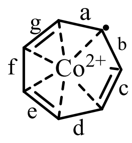	1.41109	1.41117	1.41108	1.41125	1.41112	1.41111	1.41106

Wiberg Bond Index

Without metal

Table S3. Wiberg bond index of Cyclopropenyl radical

S. No	Atom	WBI
1.	C	3.8134
2.	C	3.8143
3.	C	3.0909
4.	H	0.9582
5.	H	0.9582
6.	H	0.9431

Table S4. Wiberg bond index of Cyclobutenyl anionic radical

S. No	Atom	WBI
1.	C	3.6835
2.	C	3.6759

3.	C	3.6834
4.	C	3.6757
5.	H	0.9861
6.	H	0.9861
7.	H	0.9861
8.	H	0.9861

Table S5. Wiberg bond index of Cyclopentadienyl radical

S. No	Atom	WBI
1.	C	3.7235
2.	C	3.7248
3.	C	3.9148
4.	H	0.9635
5.	C	3.9196
6.	H	0.9634
7.	C	3.4754
8.	H	0.9593
9.	H	0.9593
10.	H	0.9662

Table S6. Wiberg bond index of Cyclohexa1-3diene cationic radical

S. No	Atom	WBI
1.	C	3.8678
2.	C	3.8673
3.	C	3.5013
4.	C	3.8678
5.	C	3.8673
6.	C	3.5013
7.	H	0.9407
8.	H	0.9407
9.	H	0.9480
10.	H	0.9407
11.	H	0.9480
12.	H	0.9407

Table S7. Wiberg bond index of Cycloheptatrienyl radical

S. No	Atom	WBI
1.	C	3.9367
2.	C	3.6429
3.	C	3.9498
4.	C	3.8095
5.	C	3.6566
6.	C	3.8299
7.	C	3.9310
8.	H	0.9662
9.	H	0.9663

10.	H	0.9661
11.	H	0.9663
12.	H	0.9663
13.	H	0.9663
14.	H	0.9662

Atom-atom overlap-weighted NAO bond order

Table S8. Atom-atom overlap-weighted NAO bond order of Cyclopropenyl radical

Atom with C	BO
C1-C2	1.2801
C2-C3	0.8582
C3-C1	0.8568

Table S9. Atom-atom overlap-weighted NAO bond order of Cyclobutenyl anionic radical

Atom with C	BO
C1-C2	1.1668
C2-C3	0.8556
C3-C4	1.1668
C4-C1	0.8555

Table S10. Atom-atom overlap-weighted NAO bond order of Cyclopentadienyl radical

Atom with C	BO
C1-C3	1.2254
C3-C7	1.0280
C7-C5	1.0273
C5-C2	1.2259
C2-C1	0.9176

Table S11. Atom-atom overlap-weighted NAO bond order of Cyclohexa1-3diene cationic radical

Atom with C	BO
C1-C2	1.2028
C2-C3	1.0307
C3-C4	1.0310
C4-C5	1.2028
C5-C6	1.0307
C6-C1	1.0310

Table S12. Atom-atom overlap-weighted NAO bond order of Cycloheptatrienyl radical

Atom with C	BO
C1-C2	1.0340
C2-C3	1.1408
C3-C5	1.1557
C5-C7	1.0226
C7-C6	1.2527
C6-C4	0.9768

C4-C1	1.2458

With metal neutral or charged

Table S13. Wiberg bond index of Cyclopropenyl radical complex

S. No	Atom	Neutral	Charged
1.	Co	1.4404	0.4719
2.	C	3.7141	3.8009
3.	C	3.7244	3.7970
4.	C	3.7184	3.7970
5.	H	0.9491	0.8969
6.	H	0.9489	0.8972
7.	H	0.9493	0.8972

Table S14. Wiberg bond index of Cyclobutenyl anionic radical complex

S. No	Atom	Neutral	Charged
1.	C	3.7701	3.8612
2.	C	3.7255	3.8613
3.	C	3.7263	3.8614
4.	C	3.7731	3.8613
5.	H	0.9792	0.9404
6.	H	0.9728	0.9404
7.	H	0.9727	0.9405
8.	H	0.9795	0.9405
9.	Co	0.9394	1.1627

Table S15. Wiberg bond index of Cyclopentadienyl radical complex

S. No	Atom	Neutral	Charged
1.	Co	0.8700	1.0809
2.	C	3.8948	3.7492
3.	C	3.8949	3.7497
4.	C	3.8958	3.7936
5.	H	0.9598	0.9136
6.	C	3.8958	3.7936
7.	H	0.9598	0.9134
8.	C	3.8943	3.6587
9.	H	0.9600	0.9087
10.	H	0.9600	0.9086
11.	H	0.9596	0.9185

Table S16. Wiberg bond index of Cyclohexa1-3diene cationic radical complex

S. No	Atom	Neutral	Charged
1.	Co	0.9960	0.7381

2.	C	3.9234	3.7750
3.	C	3.9234	3.7751
4.	C	3.9234	3.5174
5.	C	3.9234	3.7753
6.	C	3.9234	3.7749
7.	C	3.9234	3.5168
8.	H	0.9396	0.8911
9.	H	0.9396	0.8911
10.	H	0.9396	0.8993
11.	H	0.9396	0.8910
12.	H	0.9396	0.8993
13.	H	0.9396	0.8910

Table S17. Wiberg bond index of Cycloheptatrienyl radical complex

S. No	Atom	Neutral	Charged
1.	Co	1.5951	1.0855
2.	C	3.9295	3.9062
3.	C	3.8933	3.9062
4.	C	3.9295	3.9063
5.	C	3.8644	3.9062
6.	C	3.8648	3.9062
7.	C	3.9670	3.9062
8.	C	3.9670	3.9062
9.	H	0.9595	0.9202
10.	H	0.9605	0.9202
11.	H	0.9595	0.9202
12.	H	0.9589	0.9202
13.	H	0.9589	0.9202
14.	H	0.9665	0.9202
15.	H	0.9665	0.9202

Table S18. Atom-atom overlap-weighted NAO bond order of Cyclopropenyl radical complex

Atom with Co	neutral	charged
C2	0.1344	0.0542
C3	0.1446	0.0540
C4	0.1465	0.0539

Atom with C	neutral	charged
C2-C3	0.9583	1.0452
C3-C4	0.9557	1.0446
C4-C2	0.9590	1.0453

Table S19. Atom-atom overlap-weighted NAO bond order of Cyclobutenyl anionic radical complex

Atom with Co	neutral	Charged
C1	0.0221	0.1530
C2	0.1566	0.1534
C3	0.1548	0.1513

C4	0.0210	0.1510

Atom with C	neutral	Charged
C1-C2	1.0611	1.2000
C2-C3	0.8776	0.7777
C3-C4	1.0632	1.2018
C4-C1	0.9977	0.7778

Table S20. Atom-atom overlap-weighted NAO bond order of Cyclopentadienyl radical complex

Atom with Co	neutral	charged
C2	0.1175	0.1155
C3	0.1181	0.1167
C4	0.1175	0.0980
C6	0.1186	0.0999
C8	0.1170	0.1053

Atom with C	neutral	charged
C2-C3	0.5485	0.4157
C3-C6	0.5531	0.6126
C6-C8	0.5503	0.4874
C8-C4	0.5505	0.4887
C4-C2	0.5532	0.6122

Table S21. Atom-atom overlap-weighted NAO bond order of Cyclohexa1-3diene cationic radical complex

Atom with Co	neutral	charged
C2	0.0975	0.0697
C3	0.0975	0.0698
C4	0.0978	0.0595
C5	0.0977	0.0701
C6	0.0976	0.0700
C7	0.0977	0.0592

Atom with C	neutral	charged
C2-C3	0.5524	0.6146
C3-C4	0.5521	0.4767
C4-C5	0.5521	0.4767
C5-C6	0.5524	0.6146
C6-C7	0.5521	0.4765
C7-C2	0.5521	0.4766

Table S22. Atom-atom overlap-weighted NAO bond order of Cycloheptatrienyl radical complex

Atom with Co	neutral	charged
C2	0.1140	0.0822
C3	0.0197	0.0821
C4	0.1144	0.0821

C5	0.2521	0.0821
C6	0.2520	0.0822
C7	-0.0123	0.0820
C8	-0.0123	0.0820

Atom with C	neutral	charged
C2-C3	0.5660	1.1169
C3-C4	0.5656	1.1169
C4-C6	0.5667	1.1170
C6-C8	0.4622	1.1168
C8-C7	0.6668	1.1170
C7-C5	0.4623	1.1170
C5-C2	0.5663	1.1169

Electron Localization Function (ELF)

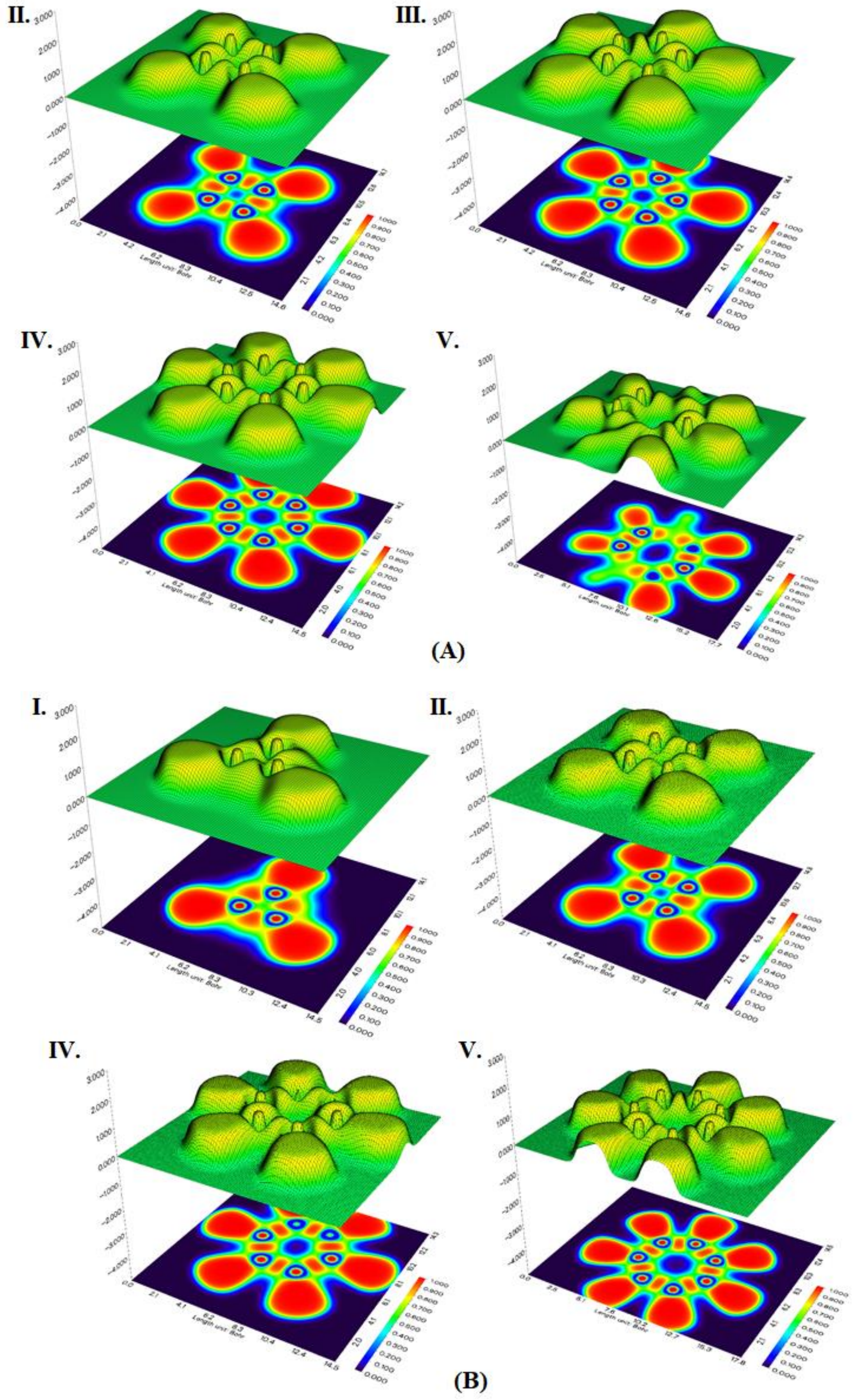

Figure S1. The 2D map of η(r) function for the molecular plane defined by the C, C and C atoms of the ring. The ELF has been calculated using the B3LYP/def2-TZVP data. (Panel A corresponds to neutral complexes and panel B corresponds to charged complexes)

Table S23. The energy values of all the Cobalt complexes with different multiplicities at B3LYP-def2-TZVP level of theory

	Co Multiplicity=3	Co^{2+} Multiplicity=3	Co Multiplicity=5	Co^{2+} Multiplicity=5
I	-1498.627992	-1497.949313	-	-
II	-1537.370289	-1537.131517	-1537.391352	-1537.105764
III	-1576.17222	-1575.426802	-1576.159398	-1575.416559
IV	-1614.681769	-1613.463931	-1614.63694	-1613.46288
V	-1653.550522	-1652.920587	-1653.518621	-

Table S24. The energy values of all the Nickel complexes with different multiplicities at B3LYP-def2-TZVP level of theory

	Ni Multiplicity=2	Ni^{2+} Multiplicity=2	Ni Multiplicity=4	Ni Multiplicity=4
I				
II	-1662.94417	-1662.69054	-1662.942272	
III	-1701.725408	-1700.973153	-1701.702784	-1700.95629
IV	-1740.230256			
V	-1779.086281	-1778.464791		

Table S25. The energy values of all the Iron complexes with different multiplicities at B3LYP-def2-TZVP level of theory

	Fe Multiplicity =2	Fe^{2+} Multiplicity= 2	Fe Multiplicity =4	Fe^{2+} Multiplicity= 4	Fe Multiplicity =6	Fe^{2+} Multiplicity= 6
I						
II	-1418.288756	-1418.073528	-1418.310418	-1418.066688	-1418.329966	-1418.048579
III	-1457.067671	-1456.36131		-1456.375187		
IV	-1495.574821	-1494.382291	-1495.608724		-1495.583814	-1494.416147
V		-1533.813985				

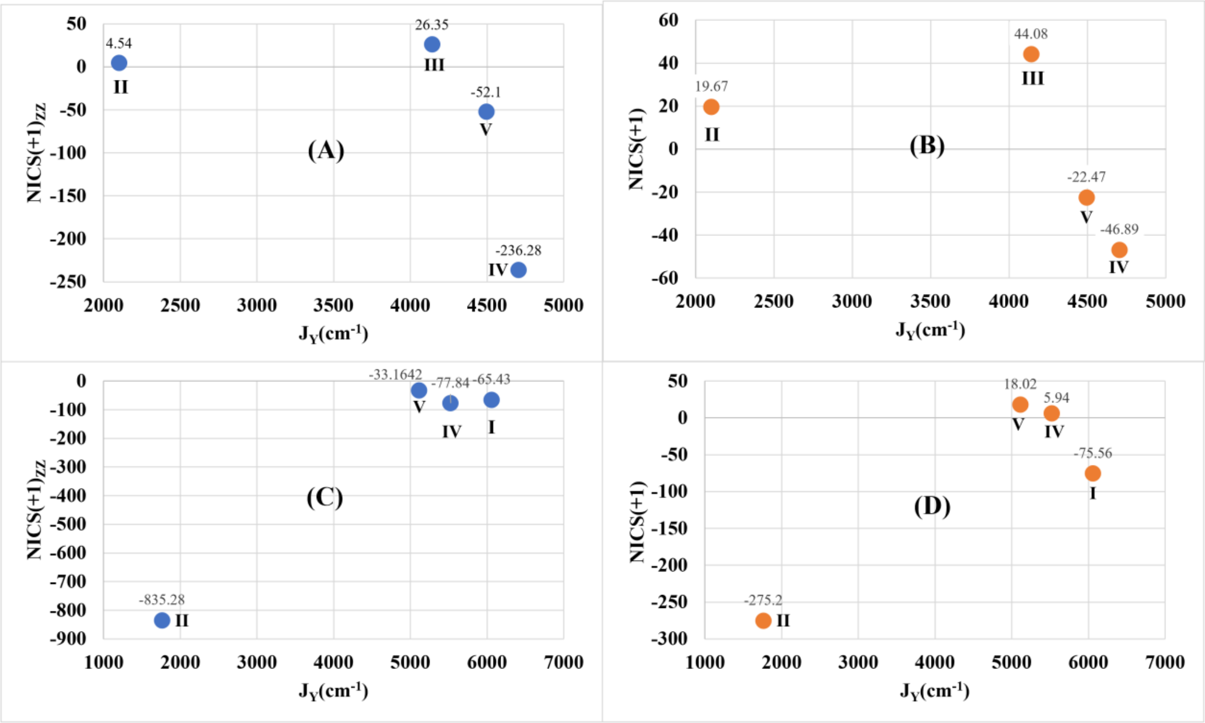

FigureS2. NICS (+1) $_{zz}$ and NICS (+1) vs *J* plot for all the complexes. (A) and (B) is for neutral complexes and (C) and (D) is for charged complexes.

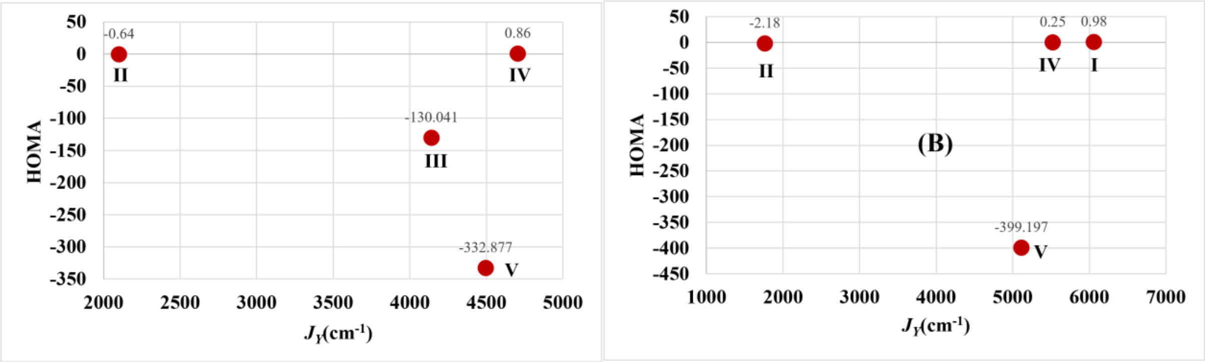

FigureS3. HOMA vs *J* plot for all the complexes. (A) is for neutral complexes and (B) is for charged complexes.

Optimized Coordinates

Without metal

I. Cyclopropenyl radical

6	-2.495321000	4.634094000	5.825533000
6	-1.388426000	5.072270000	6.367445000
6	-1.973615000	5.895960000	5.320098000
1	-3.251506000	3.870101000	5.775712000
1	-0.595550000	4.921535000	7.079258000
1	-2.399331000	6.896752000	5.380307000

II. Cyclobutenyl anionic radical

6	1.088618000	3.162026000	-1.985114000
6	0.490070000	3.053770000	-0.730185000
6	-0.854737000	2.823097000	-1.388338000
6	-0.256356000	2.931399000	-2.643416000
1	1.990175000	3.672743000	-2.316992000
1	0.774750000	3.438162000	0.246927000
1	-1.755773000	2.312161000	-1.055411000
1	-0.540635000	2.547341000	-3.620748000

III. Cyclopentadienyl radical

6	1.686314000	2.733018000	12.055149000
6	0.208552000	2.728337000	11.995154000
6	2.123704000	3.983097000	11.719602000
1	2.296127000	1.882495000	12.320786000
6	-0.207983000	3.975481000	11.624619000
1	-0.415382000	1.873935000	12.210822000
6	0.964627000	4.780189000	11.445789000
1	3.146091000	4.321005000	11.666315000
1	-1.224799000	4.306993000	11.488619000
1	0.973267000	5.820579000	11.152383000

IV. Cyclohexa 1-3diene cationic radical

6	-2.379561000	-1.174920000	4.437989000
6	-1.019310000	-1.063695000	4.447952000
6	-0.427473000	0.232857000	4.475844000
6	-1.222193000	1.415985000	4.493740000
6	-2.582443000	1.304764000	4.483763000
6	-3.174277000	0.008213000	4.455620000
1	-2.865146000	-2.141057000	4.416878000
1	-0.383201000	-1.938254000	4.435160000
1	0.652455000	0.321049000	4.483708000
1	-3.218563000	2.179315000	4.496672000
1	-4.254207000	-0.079954000	4.447342000
1	-0.736610000	2.382118000	4.515003000

V. Cycloheptatrienyl radical

6	5.668108000	6.021549000	2.514495000
6	7.082408000	6.210176000	2.541005000
6	7.775714000	7.331473000	2.985700000
6	4.676105000	6.863645000	2.909167000
6	7.300055000	8.524620000	3.508073000
6	4.801114000	8.192684000	3.470218000
6	5.934744000	8.896099000	3.722600000
1	5.343656000	5.063546000	2.120048000
1	7.678779000	5.386545000	2.169918000
1	8.857232000	7.260772000	2.912279000
1	3.660167000	6.504178000	2.794000000
1	8.041248000	9.262060000	3.788671000
1	3.864280000	8.679559000	3.715881000
1	5.792941000	9.884505000	4.148317000

With metal neutral Cobalt

I. Cyclopropenyl radical complex

27	-0.944451000	4.638054000	4.296852000

6	-2.440154000	4.548246000	5.657345000
6	-1.239061000	5.021156000	6.236775000
6	-2.023517000	5.887436000	5.437817000
1	-3.300294000	3.923697000	5.819424000
1	-0.641647000	4.977499000	7.130246000
1	-2.384224000	6.896827000	5.347526000

II. Cyclobutenyl anionic radical complex

6	1.034957000	3.303570000	-2.014891000
6	0.484074000	3.086279000	-0.713316000
6	-0.792357000	2.596646000	-1.363671000
6	-0.202169000	2.829691000	-2.644347000
1	1.998846000	3.623624000	-2.381845000
1	0.740993000	3.425348000	0.278778000
1	-1.801767000	2.435986000	-1.016845000
1	-0.514453000	2.648872000	-3.662282000
27	0.726001000	1.202340000	-1.524151000

III. Cyclopentadienyl radical complex

27	1.023942000	3.135949000	10.040605000
6	1.662349000	2.687176000	12.045507000
6	0.233080000	2.682790000	11.988026000
6	2.110151000	3.990761000	11.687717000
1	2.288481000	1.863124000	12.348330000
6	-0.192574000	3.983584000	11.594549000
1	-0.410380000	1.854643000	12.239048000
6	0.965821000	4.797581000	11.408501000
1	3.137276000	4.319259000	11.657731000
1	-1.216011000	4.305737000	11.482148000
1	0.972864000	5.845053000	11.153362000

IV. Cyclohexa 1-3diene cationic radical complex

27	-1.790280000	0.153864000	2.759221000
6	-2.403729000	-1.154013000	4.419887000
6	-0.998105000	-1.038961000	4.430876000
6	-0.394658000	0.236004000	4.457948000
6	-1.197343000	1.396048000	4.475808000
6	-2.602996000	1.280983000	4.465477000
6	-3.206397000	0.006019000	4.436736000
1	-2.866649000	-2.130310000	4.381688000
1	-0.382565000	-1.927298000	4.401115000
1	0.682832000	0.324227000	4.448937000
1	-3.218382000	2.169919000	4.461891000
1	-4.283686000	-0.081572000	4.411289000
1	-0.734229000	2.372990000	4.480362000

V. Cycloheptatrienyl radical complex

27	6.407832000	7.683594000	1.382437000
6	5.661722000	6.075743000	2.520668000
6	7.036465000	6.147100000	2.862701000
6	7.733852000	7.376112000	2.988298000
6	4.737986000	7.156246000	2.568851000
6	7.136038000	8.660281000	3.111101000
6	4.794064000	8.157505000	3.659387000
6	5.904213000	8.852534000	3.911153000
1	5.318179000	5.133952000	2.099914000
1	7.628424000	5.250534000	2.734240000
1	8.815351000	7.328301000	2.886679000
1	3.752391000	6.934111000	2.175204000
1	7.829804000	9.493481000	3.094788000
1	3.906733000	8.293769000	4.270745000
1	5.934885000	9.564827000	4.730582000

With metal charged Cobalt

I. Cyclopropenyl radical complex

27	-0.764339000	4.520399000	3.988666000
6	-2.524362000	4.622426000	5.815555000
6	-1.363397000	5.087152000	6.397370000
6	-2.126919000	5.927865000	5.615421000
1	-3.291204000	3.853708000	5.737815000
1	-0.550907000	4.948606000	7.108446000
1	-2.352220000	6.932759000	5.262712000

II. Cyclobutenyl anionic radical complex

6	1.080970000	3.247083000	-1.972586000
6	0.491031000	3.135585000	-0.729580000
6	-0.811143000	2.634106000	-1.392866000
6	-0.221264000	2.745743000	-2.635250000
1	2.009030000	3.662260000	-2.335311000
1	0.773257000	3.427856000	0.270504000
1	-1.811562000	2.424481000	-1.045840000
1	-0.574317000	2.663301000	-3.652037000
27	0.738125000	1.211941000	-1.549604000

III. Cyclopentadienyl radical complex

27	1.024366000	3.060364000	9.966046000
6	1.694183000	2.711280000	12.050066000
6	0.201157000	2.706278000	11.989179000
6	2.139096000	3.988674000	11.706405000
1	2.306319000	1.864551000	12.340357000
6	-0.222416000	3.980681000	11.609410000
1	-0.426997000	1.855812000	12.230138000
6	0.964892000	4.797950000	11.441935000
1	3.164726000	4.333838000	11.665112000
1	-1.243554000	4.319586000	11.486034000
1	0.973228000	5.846640000	11.160841000

IV. Cyclohexa 1-3diene cationic radical complex

27	-1.786806000	0.161945000	2.382147000
6	-2.393856000	-1.194451000	4.396396000
6	-1.001157000	-1.080566000	4.407317000
6	-0.394281000	0.234503000	4.531958000
6	-1.207180000	1.437212000	4.453765000
6	-2.599907000	1.323352000	4.443078000
6	-3.207872000	0.004508000	4.510759000
1	-2.879101000	-2.171098000	4.353733000
1	-0.363351000	-1.965556000	4.373733000
1	0.688188000	0.320624000	4.662623000
1	-3.237498000	2.209108000	4.435332000
1	-4.291724000	-0.086333000	4.625971000
1	-0.721641000	2.414653000	4.454422000

V. Cycloheptatrienyl radical complex

27	6.118944000	8.055337000	1.626462000
6	5.681813000	5.987372000	2.538091000
6	7.078494000	6.186491000	2.568082000
6	7.797110000	7.314924000	3.017372000
6	4.659106000	6.867191000	2.951880000
6	7.296852000	8.522947000	3.547997000
6	4.780335000	8.163207000	3.496627000
6	5.953998000	8.900383000	3.761720000
1	5.348296000	5.056234000	2.089930000
1	7.677003000	5.387959000	2.140008000
1	8.874286000	7.268269000	2.888546000
1	3.643820000	6.522932000	2.779222000
1	8.040066000	9.282005000	3.772816000
1	3.845989000	8.682294000	3.688188000
1	5.801828000	9.910544000	4.129808000
